# A pig model of acute *Staphylococcus aureus *induced pyemia

**DOI:** 10.1186/1751-0147-51-14

**Published:** 2009-03-27

**Authors:** Ole L Nielsen, Tine Iburg, Bent Aalbaek, Páll S Leifsson, Jørgen S Agerholm, Peter Heegaard, Mette Boye, Sofie Simon, Kristine B Jensen, Sophie Christensen, Karin Melsen, Anne K Bak, Elín R Backman, Mia H Jørgensen, Désirée K Groegler, Asger L Jensen, Mads Kjelgaard-Hansen, Henrik E Jensen

**Affiliations:** 1 Department of Veterinary Disease Biology, Faculty of Life Sciences, University of Copenhagen, Grønnegårdsvej 15, DK-1870 Frederiksberg C, Copenhagen, Denmark; 2 Department of Veterinary Diagnostics and Research, National Veterinary Institute, Technical University of Denmark, Bülowsvej 27, DK-1790 Copenhagen V, Denmark; 3 Department of Small Animal Clinical Sciences, Faculty of Life Sciences, University of Copenhagen, Dyrlægevej 16, DK-1870 Frederiksberg C, Copenhagen, Denmark

## Abstract

**Background:**

Sepsis caused by *Staphylococcus aureus *constitutes an important cause of morbidity and mortality in humans, and the incidence of this disease-entity is increasing. In this paper we describe the initial microbial dynamics and lesions in pigs experimentally infected with *S. aureus*, with the aim of mimicking human sepsis and pyemia.

**Methods:**

The study was conducted in anaesthetized and intravenously inoculated pigs, and was based on bacteriological examination of blood and testing of blood for IL-6 and C-reactive protein. Following killing of the animals and necropsy bacteriological and histological examinations of different organs were performed 4, 5 or 6 h after inoculation.

**Results:**

Clearance of bacteria from the blood was completed within the first 2 h in some of the pigs and the highest bacterial load was recorded in the lungs as compared to the spleen, liver and bones. This probably was a consequence of both the intravenous route of inoculation and the presence of pulmonary intravascular macrophages. Inoculation of bacteria induced formation of acute microabscesses in the lungs, spleen and liver, but not in the kidneys or bones. No generalized inflammatory response was recorded, i.e. IL-6 was not detected in the blood and C-reactive protein did not increase, probably because of the short time course of the study.

**Conclusion:**

This study demonstrates the successful induction of acute pyemia (microabscesses), and forms a basis for future experiments that should include inoculation with strains of *S. aureus *isolated from man and an extension of the timeframe aiming at inducing sepsis, severe sepsis and septic shock.

## Background

Sepsis constitutes an important cause of morbidity and mortality in humans, and the incidence of this disease-entity is increasing. At present, 660,000 cases of sepsis occur in the USA each year and combined with the high mortality, this ranks sepsis as a leading cause of death in this country. Staphylococci, including methicillin resistant *Staphylococcus aureus *(MRSA), have become the most frequently isolated bacteria in nosocomial infections giving rise to more than 50% of the cases [1]. Similar observations have been made in other countries, including Denmark [2,3]. Staphylococcal seeding to e.g. endocardium, skeleton and lungs resulting in the development of pyemic lesions (i.e., infective endocarditis, pyogenic osteomyelitis and lung abscesses) are serious complications to sepsis [4-6].

Models of bacteraemia, sepsis and pyemia caused by *S. aureus *have been established primarily in small laboratory animals (mice, rats, guinea pigs, and rabbits), while studies of experimental blood stream infection with *S. aureus *in pigs are few. Some of these studies in pigs aimed at the characterization of pulmonary intravascular macrophages such as the study by Winkler [7], some were models of prosthetic device infections exemplified by that reported by Paget *et al. *[8], and some investigated the pulmonary haemodynamics and function such as those by Walther *et al. *[9,10]. A few extensive studies modelling the pathogenesis of human sepsis and the ensuing shock have been performed with group A streptococci in pigs [11-14], but most of this type of research has been performed with Gram-negative bacteria or endotoxin [15].

Pigs may spontaneously develop pyemia and based on records from the *post mortem *meat inspection, approximately 125,000 pigs (0.4% of the total number of slaughtered pigs) are each year diagnosed with pyemia in Denmark (Ministry of Food, Agriculture and Fisheries, Danish Veterinary and Food Administration, 2007, unpublished data). In a study of pyemic lung lesions in pigs, *S. aureus *was found in monoculture in 46% of the cases [16]. Pig farming is a risk factor for nasal *S. aureus *colonization in man and sequence typing and phylogenetic comparisons of isolates have suggested a high rate of strain exchange between pigs and pig farmers [17]. Similar studies on MRSA showed an increased prevalence rate of nasal colonization in persons in contact with pigs [18] and infections in humans with MRSA have been related to a domestic animal source that included pigs [19,20]. The reported pig-associated subtype of MRSA, i.e. clonal complex (CC) 398, also has been identified in pigs and man from Denmark [21], and the paper draws similar epidemiological and zoonotical conclusions as reported by others.

The aim of the present study was to study the initial microbial dynamics and lesions in pigs inoculated intravenously with *S. aureus*. With the increasing use of the pig in biomedical research, a model of *S. aureus *sepsis and pyemia could prove useful to the study of the disease in man, but also to the study of the disease in pigs, as spontaneous generalized *S. aureus *infections are of major concern in both species.

## Methods

### Animals and housing

Nine clinically healthy Yorkshire-Landrace-Duroc crossbreed pigs (nos. 1–9), body weight (BW) of 20–25 kg corresponding to 9–10 weeks of age, were obtained from a commercial pig herd. The pigs were allowed to acclimatize for 5–10 days before entering the trial. Food was withdrawn 12 h before the start of the experiment, and immediately before the start the pigs underwent a clinical examination and measurement of body temperature, to secure absence of clinical signs of disease.

### Experimental design

The pigs were sedated by intramuscular injection of a solution containing a mixture of zolazepam, tiletamine, xylazine and ketamine (0.83 mg/kg BW of each of the drugs), and butorphanol (0.17 mg/kg BW). A catheter (22 G) was then inserted in the right ear vein and used for infusion of anaesthetics, which consisted of a solution containing a mixture of xylazine (1 mg/mL), ketamine (2 mg/mL), butorphanol (0.1 mg/mL) and guaifenesine (48 mg/mL).

A catheter (22 G) was inserted in the left ear vein and eventually used for the administration of bacteria or mock followed by flushing with 10 mL sterile isotonic saline. After this procedure the catheter was removed. Another catheter (diameter of 2.6 mm) was surgically inserted into the left external jugular vein, adjusted to sit in the bi-jugular trunk, fixed to the skin with stitches, and flushed with 10 mL sterile isotonic saline followed by 2 mL of sterile 18 EI heparin solution. This catheter was used for blood sampling. The samples were secured free of heparin solution by discarding the first 5 mL of blood. All surgical procedures, insertion of catheters, injections, withdrawal of blood, aliqotation of blood etc. were performed strictly aseptically using 70% ethanol as disinfectant.

Eight pigs (nos. 1–6, 8 and 9) were inoculated with *S. aureus*, and one (no. 7) was mock-inoculated with sterile isotonic saline. Examination of blood included bacteriology on full blood and measurement of IL-6 in plasma and C-reactive protein in serum. The blood samples were taken at regular intervals until killing of the pigs 4 h after inoculation (PI) (nos. 1–4 and 7), 5 h PI (nos. 6 and 9) or 6 h PI (nos. 5 and 8), and included samples taken 2 min before inoculation (-2 min). Sampling timepoints for bacteriology were -2, 2, 30, 60, 120, 240, 300 and 360 min as indicated in Table 1, and timepoints for the testing of IL-6 and C-reactive protein were -2, 30, 60, 90, 120, 150, 180, 210, 240, 270, 300, 330 and 360 min. The *post mortem *examination included bacterial culture from organs, histopathology, and fluorescent *in situ *hybridisation for bacteria. The study was conducted in accordance with the EU directive 86/609 and the Danish Animal Experimentation Act.

**Table 1:** Viable count of *Staphylococcus aureus *in blood

			Blood sampling timepoints (min)							
			
Pig no.	Innoculation^a^	Pig killed (h PI^b^)	-2^c^	2^d^	30	60	120	240	300	360
1	*S. aureus*	4	0	2600^e^	29	13	0	0		

2	*S. aureus*	4	0	2200	25	8	5	0		

3	*S. aureus*	4	0	6500	47	10	2	4		

4	*S. aureus*	4	0	400	47	11	0	0		

5	*S. aureus*	6	0	850	50	18	2	1	NT^f^	-^g^

6	*S. aureus*	5	0	1100	100	57	14	4	2	

8	*S. aureus*	6	0	480	34	12	5	2	NT	0

9	*S. aureus*	5	0	520	88	46	21	2	1	

Mean	*S. aureus*	4 – 6	0	1800	53	22	6	2		

7	Mock	4	0	0	0	0	0	0		

^a ^1 mL of 10^8 ^CFU were inoculated intravenous per kg of body weight and 1 control pig was injected with and equal volume of sterile isotonic saline (mock)

^b ^PI, after inoculation

^c ^"-2" indicates that blood was collected 2 min before inoculation

^d ^Blood was collected 2 min PI

^e ^Numbers indicate viable counts per ml of blood

^f ^NT, not tested

^g ^Result missing

### Staphylococcus aureus suspension

*Staphylococcus aureus*, isolate no. S54F9 was obtained from a chronic embolic pulmonary abscess in a Danish slaughter pig (Department of Veterinary Pathobiology journal no. 36444). The isolate was identified using Api ID 32 Staph (Biomerieux, Inc., Marcy-l'Etoile, France) and was propagated in 100 mL of Luria-Bertani (LB) broth [22] for 18 h at 37°C, sedimented by centrifugation and re-suspended in sterile isotonic saline. The viable count was determined by counting the number of colonies formed on LB agar medium inoculated with 10 μL volumes of ten fold dilutions and incubated at 37°C for 24 h. The suspension was diluted with sterile isotonic saline to obtain a suspension containing 10^8 ^colony-forming units (CFU)/mL. This was used for intravenous inoculation at a dose of 10^8 ^CFU/kg BW.

As part of another study, the strain was typed by tandem repeat analysis of the staphylococcal protein A (*spa*) gene, a standard method for molecular typing of *S. aureus *[23]. The *spa *type observed in this strain (t1333) is one of the two most common types among porcine clinical *S. aureus *isolates in Denmark and is associated with clonal complex 30 according to the classification based on multi-locus sequence typing (Bent Aalbaek and Luca Guardabassi, unpublished data).

### Bacteriological examination of blood and organs

Heparin-stabilized blood samples of 10 mL were taken aseptically and kept at 5°C for a maximum of 4 h until being processed. Blood in volumes of 1 mL and 1 mL of decimal dilutions (using sterile isotonic saline) were added to empty Petri dishes and mixed with melted LB agar medium. Viable count was determined after incubation for 48 h at 37°C and presented as counts/mL blood.

Quantitative bacteriological examination was performed on the lung (left diaphragmatic lobe), spleen (dorsal half) and liver (left lateral lobe) from all nine pigs upon euthanasia. In addition, bone tissue from the metaphysis/physis region of the left femur was cultured in 4 pigs (nos. 5, 6, 8, 9). The samples were kept at 5°C for a maximum of 12 h before being processed. Approximately 1 g of tissue was removed aseptically from the organs, cut into minor pieces with a scalpel, weighed and homogenized in 9 mL of sterile isotonic saline using a stomacher. Ten fold dilutions in sterile isotonic saline of the homogenized tissues were prepared. From each of these preparations 10 μL were inoculated on the surface of an LB agar medium and incubated for 48 h at 37°C before counting the colonies. The counts/g tissue were then calculated. Colony morphology was evaluated and representative colonies were subcultured on blood agar (Blood agar base, CM55; Oxoid, Basingstoke, Hampshire, England) containing 5% sterile bovine blood and phenotypically characterized using Api ID 32 Staph.

### Assays for plasma IL-6 and serum C-reactive protein

Plasma was generated by centrifugation of ethylenediaminetetraacetic acid (EDTA) stabilised blood sampled in endotoxin free vials. Centrifugation was performed immediately after blood had been collected. The plasma samples were kept at 5°C for maximum 1 h before storing at -80°C. The IL-6 content was determined in plasma (diluted 1/2) by an R & D Systems DuoSet ELISA (R & D Systems, Abingdon, UK, catalog no. DY686), using ELISA plates from Nunc (Roskilde, Denmark, type: Macrosorp), and using goat anti porcine IL-6 for coating (0.8 μg/mL in PBS), biotinylated goat anti porcine IL-6 (0.1 μg/mL in PBS with 1% bovine serum albumin (BSA), Sigma St. Louis, MO, catalog no. A2153) and peroxidase-conjugated streptavidin (from the DuoSet kit, diluted 1/200) for detection, and finally TMB Plus from Kem-En-Tec (Taastrup, Denmark) as chromogen. A standard preparation of recombinant porcine IL-6 (from the DuoSet kit) was applied in double determination as a two-fold dilution row from 8000 pg/mL to 125 pg/mL. Two wells were used for buffer controls. Sample values for IL-6 were calculated from the curve fitted to the readings of the standard (using Ascent software v. 2.6).

Serum was generated by centrifugation of blood samples left to coagulate for no longer than 1 h at 22°C in plain, endotoxin free vials. The serum samples were kept at 5°C for maximum 1 h before storing at -80°C. The C-reactive protein (CRP) content in serum was determined on the ADVIA 1650 (Bayer), using a procedure that included loading undiluted serum into the machine [24].

### Post mortem examination and histopathology

The post mortem examination included sagittal sections of the bones. Tissue samples of the lungs (dorsal part of the diaphragmatic lobes), the spleen, the liver, the kidneys, and the metaphysis/physis region of the right femur, tibia, radius, ulna, sacral bone, thoracic vertebrae nos. 8 and 9 plus the costochondral junction of the 8^th ^and 9^th ^ribs were fixed for 24 h in PBS buffered 4% formaldehyde. After fixation, bone tissues were decalcified for 6 days in a solution of EDTA and sodium hydroxide (280 g EDTA and 30 g NaOH dissolved in 2000 mL of water). The tissue specimens were then processed through graded concentrations of ethanol and xylene, embedded in paraffin wax, cut at 3–5 μm, rehydrated, and stained with haematoxylin and eosin (HE) [25]. Tissue sections for *in situ *hybridisation were mounted on Super Frost Plus glass slides (Gerhard Menzel, Braunschweig, Germany) and processed as stated below.

### Fluorescent in situ hybridisation

Fluorescent *in situ *hybridisation (FISH) was applied on selected tissue sections using an Alexa 555 5'-labeled oligonucleotide probe (EUB 338) targeting a 16S rRNA sequence specific for the Domain Bacteria [26]. The procedure was modified after Boye *et al. *[27] and included a 10 min pre-treatment at 20°C of the sections with 3 mg/mL of lysozyme (cat. no. L-6876, Sigma Aldrich, USA) dissolved in a Tris/EDTA-buffer (100 mM Tris and 50 mM EDTA (pH 6.5)). The sections were rinsed in water and hybridised for 16 h in a moist chamber at 40°C with 5 ng/mL of probe dissolved in a hybridisation buffer (700 mM NaCl, 100 mM Tris (pH 8) and 0.1% sodium dodecyl sulphate (SDS)). Washing of the sections was performed 2 times with 2 × standard saline citrate (SSC) for 1 min, with hybridisation buffer prewarmed to 45°C for 20 min, and finally 2 times with 2 × SSC for 1 min.

## Results

### Bacteriological examination of blood and organs

The viable counts obtained from blood and tissue samples are given in Table 1 and 2. All colonies had a morphology identical to that of the inoculation strain. Representative isolates showed the same reaction pattern in API Staph as the strain used for inoculation.

**Table 2:** Viable count of *Staphylococcus aureus *in organs

			Organs^c^			
			
Pig no.	Innoculation^a^	Pig killed (h PI^b^)	Lung	Spleen	Liver	Metaphysis/physis region of bone
1	*S. aureus*	4	140000	2800	93000	NT^d^

2	*S. aureus*	4	130000	18000	7700	NT

3	*S. aureus*	4	290000	11000	3800	NT

4	*S. aureus*	4	110000	2700	9600	NT

5	*S. aureus*	6	78000	34000	3300	1400

6	*S. aureus*	5	26000	43000	34000	2700

8	*S. aureus*	6	50000	16000	1200	730

9	*S. aureus*	5	47000	63000	15000	4300

Mean	*S. aureus*	4 – 6	110000	24000	21000	2300

7	Mock	4	0	0	0	NT

^a ^1 mL of 10^8 ^CFU were inoculated intravenous per kg of body weight and 1 control pig was injected with and equal volume of sterile isotonic saline (mock)

^b ^PI, after inoculation

^c ^Numbers indicate viable counts per g of tissue

^d ^NT, not test

### Plasma IL-6 and serum C-reactive protein

On each of the time points tested, the content of IL-6 was under the detection limit of the assay (250 pg/mL). Also, CRP measurements did not reveal any significant increases or variations.

### Gross pathology, histopathology and FISH

All pigs showed atelectasis of the dorsal part of the diaphragmatic lobes. This probably was related to the dorsal recumbency of the pigs during anaesthesia, and thus a result of the experimental design.

**Figure 1:**
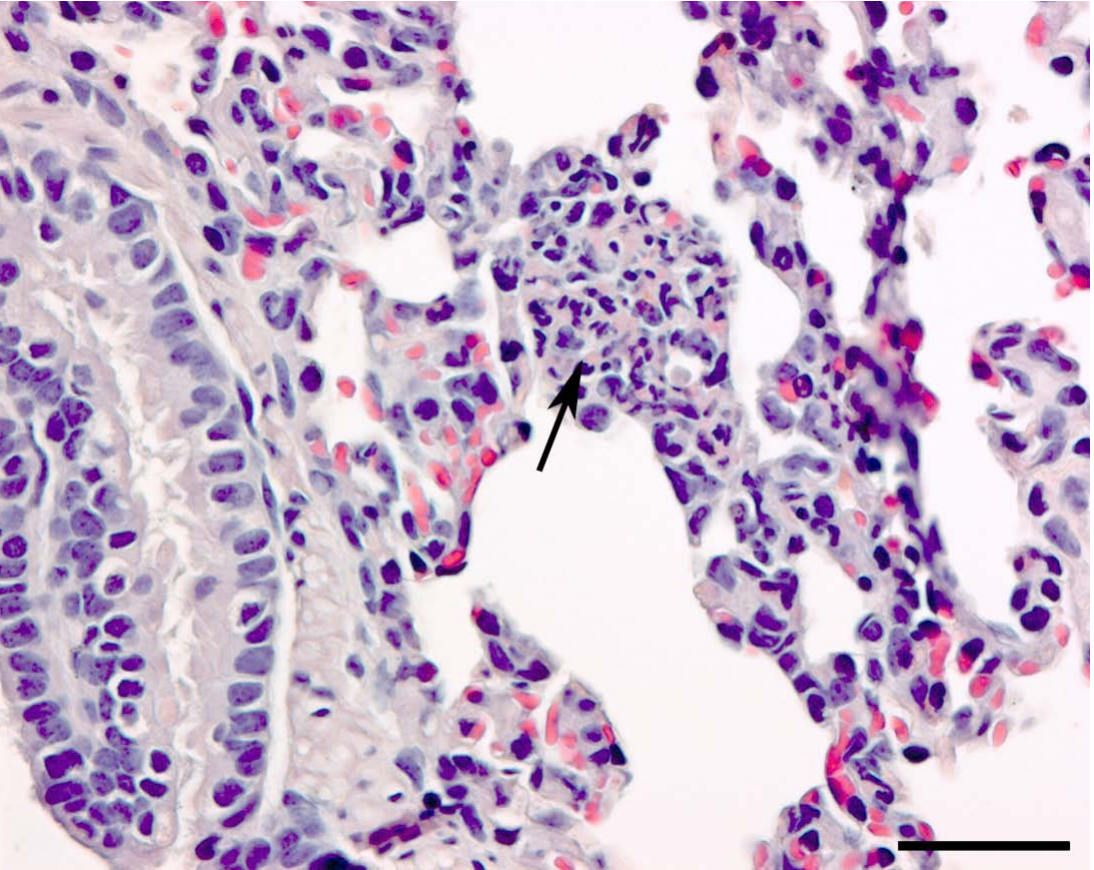
**Microabscess in the lung**. Section of lung from a *Staphylococcus aureus *infected pig killed 6 h after inoculation (pig no. 5) showing a microabscess (arrow) located to an alveolar septum. Haematoxylin- and eosin stain. Bar = 50 μm.

**Figure 2:**
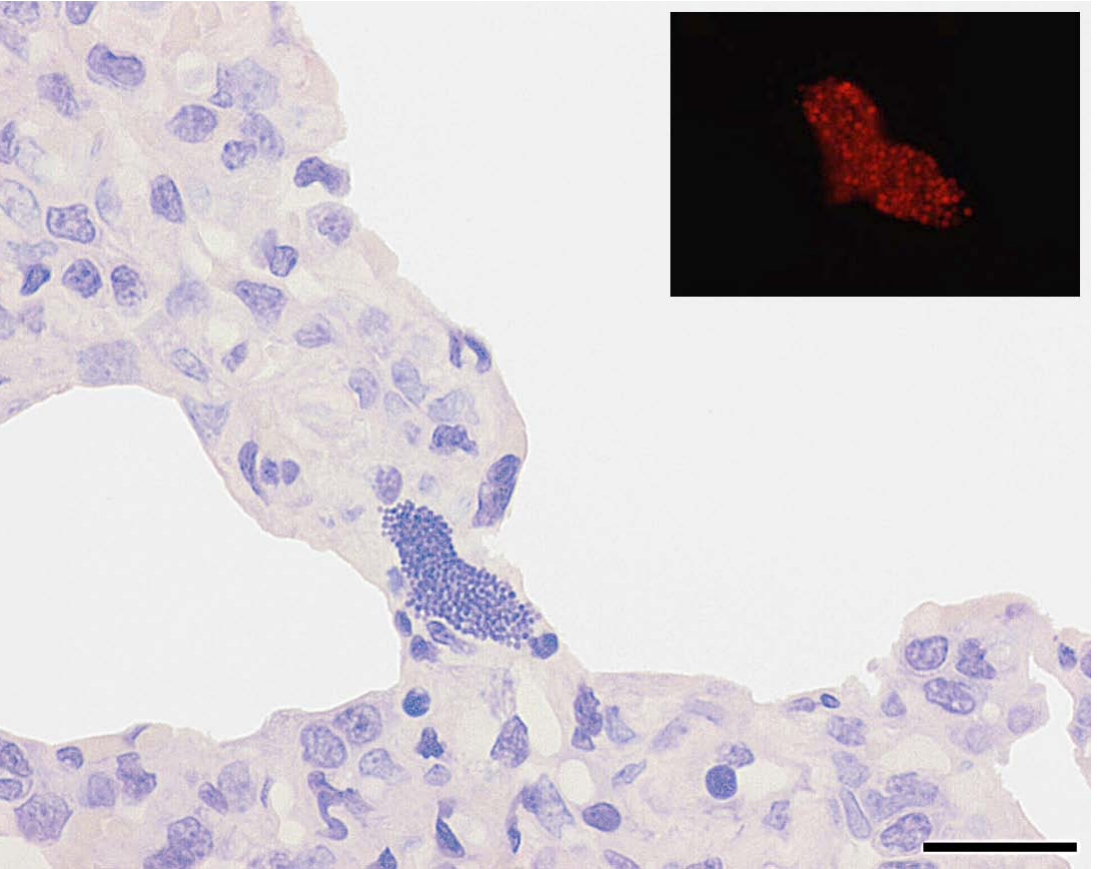
**Bacterial colony in the lung**. Section of lung from a *Staphylococcus aureus *infected pig killed 5 h after inoculation (pig no. 6) showing a bacterial colony without any inflammatory reaction and identified by fluorescent *in situ *hybridisation (insert). The *in situ *hybridisation was performed first and the bacterial colony photographed. Then the section was stained with haematoxylin and the same colony identified and a new photo taken. Bar = 20 μm.

The HE stained section of the lungs revealed presence of acute microabscesses (Figure 1) and aggregates of spherical, basophilic organisms in three of the four *S. aureus*-inoculated pigs killed 5 or 6 h PI (nos. 5, 6, 8). These aggregates were identified as bacterial colonies by FISH and bacterial colonies were often present without any ensuing inflammatory reaction (Figure 2). Lung lesions were absent in all *S. aureus*-inoculated pigs killed 4 h PI, one pig killed 5 h PI and the mock-inoculated pig.

Acute microabscesses were present in the marginal zone of the spleen (the zone between the red and white pulpa) in the *S. aureus*-inoculated animals killed 5 or 6 h PI (nos. 5, 6, 8, 9). In two of the *S. aureus*-inoculated pigs killed 4 h PI (nos. 1, 3) neutrophils seemed to accumulate in the marginal zone without forming true microabscesses. The remaining pigs (two *S. aureus*-inoculated and the mock-inoculated) were without histological lesions. In the liver, acute microabscesses were detected in pigs from the staphylococcus group killed 4 h PI (nos. 1, 2, 4), 5 h PI (nos. 6, 9), and 6 h PI (no. 8). The rest of the pigs, including the mock-inoculated, were without histological lesions. The kidneys and the metaphyses of all bones from both inoculated and control animals had no lesions.

## Discussion

The quantitative bacteriological examination of blood and tissues (Table 1 and 2) showed that blood samples taken 2 min after intravenous injection of *S. aureus *contained an initial mean viable count of 1800 CFU/mL. The subsequent blood samples showed decreasing numbers of bacteria reaching a mean of 2 CFU/mL 4 h PI and with 3 animals being culture negative. These results reflect both dilution of the inoculated bacteria within the blood compartment and clearance.

In the organs, which were examined 4, 5 or 6 h PI, mean viable counts largely exceeded the initial viable count obtained in the blood indicating a considerable capacity of the organs for withholding bacteria from the circulation. The mean viable count per g of spleen and liver tissue were of the same magnitude, 24,000 CFU/g and 21,000 CFU/g, respectively in contrast to a higher mean viable count from lung tissue, 110,000 CFU/g, suggesting that the porcine lung has a high capacity for retaining bacteria from the circulation. This finding is in agreement with previous reports [28,29] and is closely linked to the clearing action of pulmonary intravascular macrophages (PIM), present in swine and many other animal species but not in man [7]. When comparing the content of bacteria in different organs, the volume of blood and the blood-load of bacteria entering the organs should be taken into account [29]. Thus, the lungs will receive the total volume of blood and load of bacteria (total right ventricular outflow) whereas the spleen, for example, will only receive a fraction of the blood and only bacteria that is not withheld by the lungs or other organs. Also, proliferation or destruction of the bacteria within the organs would influence the level. Thus destruction of bacteria in the lungs could explain the seemingly lower levels in the lungs of pigs examined 5 and 6 h PI as opposed to pigs examined 4 h PI. The mean viable count from the metaphysis/physis region was 2300 CFU/g, the lowest recorded, but compared to the 0 – 2 CFU/mL present in the blood still indicates some capacity for the retention of bacteria by bone tissue.

Plasma IL-6 was not detected in any of the pigs and no increase in serum CRP was observed. IL-6 together with IL-1 and tumor necrosis factor-α (TNF-α) are some of the major proinflammatory cytokines produced in monocytes and other cells as an immediate response to infection and other stimuli. The cytokines have a range of local and systemic effects, including the recruitment of neutrophils and the induction of acute phase proteins from the liver. The systemic effects rely on the presence of cytokines in the blood and their presence is linked to a range of different factors. For example, endotoxin caused production of IL-1, IL-6 and TNFα within 1–5 h in cellular *in vitro *assays, whereas Gram-positive toxins induced a peak response of lymphotoxin-α and interferon-γ 50–75 h after challenge [30]. Infusion of endotoxin to pigs caused TNF-α and IL-6 to peak in the blood 1–4 h later [31]. In experimental aerogenous infection studies in pigs with the Gram-negative pulmonary pathogen *Actinobacillus pleuropneumoniae*, blood IL-6 was detected within the first 10–14 h PI [32,33]. Increase in blood CRP has been demonstrated in several studies as reviewed by Petersen et al. [34]. The absence of a systemic IL-6 and CRP response in our study could have a variety of causes, including the short duration of the experiment and the bacterial strain used. However, IL-6 was detected in the blood only 1 h after the intravenous inoculation of *S. aureus *in mice [35] and was produced in response to *in vitro *challenge of human endothelial cells by *S. aureus *[36]. Also, TNF-α was detected in blood only 3 h after the intravenous inoculation of serogroup A streptococci in pigs [13].

The histological examination revealed presence of acute microabscesses and bacterial colonies while evidence of thrombosis was absent. Microabscesses were seen in all *S. aureus*-inoculated animals except for one pig (no. 3) and were detected in the lung, spleen and liver, but not in the kidney and the metaphysis/physis of bones. These lesions represent acute pyemia. *S. aureus *colonies were present in only the lungs of 3 *S. aureus*-inoculated pigs. The bacterial colonies may represent trapping of bacterial emboli or local proliferation.

The presence of microabscesses in the marginal zone of the spleen has been linked to sepsis [37]. Presence of microabscesses in the lungs and the liver probably reflects the presence of PIM in the lungs and Kupffer's cells in the liver [38]. Naturally occurring pyemia in pigs is often associated with lesions in the lungs and the skeleton (Ministry of Food, Agriculture and Fisheries, Danish Veterinary and Food Administration, 2007, unpublished data). Frequently isolated bacteria from lung lesions are *S. aureus *[16] and *Arcanobacterium pyogenes *from skeleton abscesses [39]. Bacteria, including *S. aureus *are isolated from cases of osteomyelitis, and different predisposing factors, including the presence of receptors to bone surface proteins in *S. aureus*, have been suggested to explain the frequent occurrence of acute osteomyelitis localized to the metaphysial or the equivalent epiphysial regions [39]. The lack of osteomyelitis in our study could be a result of the short timeframe of the study or the rather light colonization of the skeleton.

## Conclusion

In conclusion, we were able to induce acute pyemia (the formation of acute microabscesses) in pigs 4 to 6 h after the intravenous inoculation of *S. aureus*. Microabscesses were present in the lungs, spleens and livers, but not in the kidneys or bones. Presence of IL-6 or increased levels of CRP in the blood were not seen and a septic stage, defined by the presence of these biomarkers [40], was thus not reached. Future experiments should include inoculation with strains of *S. aureus *isolated from man and an extension of the timeframe aiming at inducing sepsis, severe sepsis and septic shock, thus modelling the human disease syndromes in pig.

## Competing interests

The authors declare that they have no competing interests.

## Authors' contributions

OLN, TI, PSL, JSA and HEJ designed the study, participated in the execution of the study and supervised the histopathology. PH, ALJ and MK-H contributed substantially to designing the study. BA performed the bacteriology, including characterization of the *S. aureus *strain, preparation of the inoculum and culture from blood and organs. SS, KBJ, SC, KM, AKB, ERB, MHJ, DKG and MB made substantial contributions to the acquisition, analysis and interpretation of data. PH performed the blood analysis for IL-6. MK-H performed the blood analysis for C-reactive protein. OLN drafted the manuscript, which was reviewed and commented by TI, PSL, JSA and HEJ. All authors read and approved the final manuscript.
